# Supplementary material for: The economic burden of loiasis: A comprehensive cost-of-illness analysis of regionally representative, individual-level data from rural Gabon
Source: PLoS One. 2026 Feb 23;21(2):e0340689. doi: 10.1371/journal.pone.0340689 (PMC12928485; doi:10.1371/journal.pone.0340689)
Supplement: S1 Table — (DOCX) [file pone.0340689.s001.docx]

**S1 Table. Construction and definition of core variables**

| **Variable** | **Description** |
| --- | --- |
| *Loa loa* | A binary variable (0 = No, 1 = Yes) telling whether the participant is loiasis positive or negative. Participants reporting eye worm migration or harboring loiasis microfilariae were defined as loiasis positive. |
| Age groups | 5 binary variables (0 = No, 1 = Yes) that take the value 1 if the participant belongs to one of the following age groups: 1) 18 to 25 years old; 2) 26 to 35 years old; 3) 36 to 45 years old; 4) 46 to 55 years old; 5) 56 to 65 years old. |
| Female | A binary indicator (0 = No, 1 = Yes) that takes the value 1 if the respondent is female. |
| Work in the forest | A binary indicator (0 = No, 1 = Yes) that takes the value 1 if the respondent had some activities in the forest during the month prior to the interview. |
| Malaria infection | A binary indicator (0 = No, 1 = Yes) that takes the value 1 if the respondent had malaria during the 4 weeks prior to the interview. |
| Education level | 4 binary variables (0 = No, 1 = Yes) that take the value 1 if the respondent completed a given school level or has no education: 1) No education; 2) Primary education; 3) Secondary education; 4) Other education. |
| Wealth index | 5 binary variables (0 = No, 1 = Yes) that take the value 1 if the respondent belongs to a given wealth category: 1) Very poor; 2) Poor; 3) Middleclass; 4) Rich; 5) Very rich. |
| Village fixed effects | A spatial indicator, namely village fixed effects. |
