# Supplementary material for: The economic burden of loiasis: A comprehensive cost-of-illness analysis of regionally representative, individual-level data from rural Gabon
Source: PLoS One. 2026 Feb 23;21(2):e0340689. doi: 10.1371/journal.pone.0340689 (PMC12928485; doi:10.1371/journal.pone.0340689)
Supplement: S2 Table — (DOCX) [file pone.0340689.s002.docx]

**S2 Table. Summary statistics of core variables**

| **Variable** | **Mean** | **Median** | **SD** | **Min** | **Max** | **Obs** |
| --- | --- | --- | --- | --- | --- | --- |
|  | (1) | (2) | (3) | (4) | (5) | (6) |
| Sociodemographics | | | | | | |
| Age | 42.72 | 43.00 | 14.08 | 18 | 65 | 1269 |
| Female | 0.52 | 1.00 | 0.50 | 0 | 1 | 1269 |
| Household size | 5.07 | 4.85 | 3.30 | 1 | 20 | 1269 |
| No education | 0.06 | 0.00 | 0.24 | 0 | 1 | 1269 |
| Primary school | 0.35 | 0.00 | 0.48 | 0 | 1 | 1269 |
| Secondary school | 0.58 | 1.00 | 0.49 | 0 | 1 | 1269 |
| Other education | 0.02 | 0.00 | 0.13 | 0 | 1 | 1269 |
| Insurance coverage | 0.78 | 1.00 | 0.41 | 0 | 1 | 1269 |
| Per capita income | 68.73 | 33.46 | 93.12 | 1 | 535 | 524 |
| Q1 | 5.40 | 5.32 | 2.03 | 1 | 9 | 105 |
| Q2 | 15.09 | 14.64 | 3.71 | 9 | 21 | 103 |
| Q3 | 32.75 | 33.46 | 6.40 | 22 | 42 | 110 |
| Q4 | 72.03 | 72.35 | 17.59 | 43 | 105 | 106 |
| Q5 | 226.58 | 195.17 | 107.49 | 110 | 535 | 100 |
| *Prevalence of loiasis* | | | | | | |
| History of eye worm | 0.34 | 0.00 | 0.47 | 0 | 1 | 1269 |
| Microscopy positive | 0.24 | 0.00 | 0.43 | 0 | 1 | 1269 |
| Loiasis positive | 0.47 | 0.00 | 0.50 | 0 | 1 | 1269 |

Notes: Loiasis positivity is defined by either having a history of eye worm migration or by the confirmed presence of *Loa loa* microfilaremia. The following variables are binary indicators (0 = No, 1 = Yes) that classify whether participants belong to a category or not: Female, No education, Primary school, Secondary school, Other education, Insurance coverage. Q1 to Q5 stand for per capita income quintiles 1 (poorest 20% of households) to 5 (richest 20% of households). All expenditure values are in US dollars. “SD” refers to standard deviation, “Obs” refers to the number of observations.
