# Supplementary material for: The economic burden of loiasis: A comprehensive cost-of-illness analysis of regionally representative, individual-level data from rural Gabon
Source: PLoS One. 2026 Feb 23;21(2):e0340689. doi: 10.1371/journal.pone.0340689 (PMC12928485; doi:10.1371/journal.pone.0340689)
Supplement: S3 Table — (DOCX) [file pone.0340689.s003.docx]

**S3 Table. Summary statistics on the utilization of healthcare services**

| **Variable** | **Mean** | **Median** | **SD** | **Min** | **Max** | **Obs** |
| --- | --- | --- | --- | --- | --- | --- |
|  | (1) | (2) | (3) | (4) | (5) | (6) |
| *Healthcare utilization* | | | | | | |
| Visits outpatient - quarter | 0.06 | 0.00 | 0.23 | 0 | 1 | 1269 |
| At least 1 day inpatient - quarter | 0.04 | 0.00 | 0.20 | 0 | 1 | 1269 |
| Out of pocket expenditures - month | 9.46 | 0.00 | 56.30 | 0 | 1698 | 1269 |
| Conditional expenditures - month | 35.53 | 16.61 | 104.86 | 0 | 1698 | 338 |

Notes: ‘Visits outpatient – quarter’ and ‘At least 1 day inpatient - quarter’ are binary indicators that take the value 1 if the person visited outpatient facility/was at least once inpatient. The reference period of these variables relates to the last 3 months prior to the survey. All expenditure values are in US dollars. “SD” refers to standard deviation, “Obs” refers to the number of observations.
