# Supplementary material for: The economic burden of loiasis: A comprehensive cost-of-illness analysis of regionally representative, individual-level data from rural Gabon
Source: PLoS One. 2026 Feb 23;21(2):e0340689. doi: 10.1371/journal.pone.0340689 (PMC12928485; doi:10.1371/journal.pone.0340689)
Supplement: S5 Table — (DOCX) [file pone.0340689.s005.docx]

**S5 Table. Alternative GLM estimates: Reweighting by different moment conditions**

| **Variable** | **Main** | **Variance** |
| --- | --- | --- |
|  | (1) | (2) |
| Direct medical costs | 9.17  (29.03) | 9.17  (29.03) |
| Direct non-medical costs | 10.59  (4.29)*** | 10.59  (4.29)*** |
| Indirect costs | 20.18  (12.69)* | 20.18  (12.69)* |
| Observations | 1269 | 1269 |

Notes: Estimates refer to marginal effects and are obtained from a two-step process involving entropy balancing (step 1) and GLM (step 2). GLM refers to Generalized Linear Models. All expenditure values are in US dollars. Robust standard errors were used and are depicted in parentheses. */**/*** denote significance levels at 10/5/1 percent respectively.
