# Supplementary material for: The economic burden of loiasis: A comprehensive cost-of-illness analysis of regionally representative, individual-level data from rural Gabon
Source: PLoS One. 2026 Feb 23;21(2):e0340689. doi: 10.1371/journal.pone.0340689 (PMC12928485; doi:10.1371/journal.pone.0340689)
Supplement: S6 Table — (DOCX) [file pone.0340689.s006.docx]

**S6 Table. Alternative GLM estimates: Covariate specifications**

| **Variable** | **Main** | **Alternative** |
| --- | --- | --- |
|  | (1) | (2) |
| Direct medical costs | 9.17  (29.03) | 7.81  (28.54) |
| Direct non-medical costs | 10.59  (4.29)*** | 10.07  (4.08)*** |
| Indirect costs | 20.18  (12.69)* | 19.69  (12.24)* |
| Observations | 1269 | 1269 |

Notes: Estimates refer to marginal effects and are obtained from a two-step process involving entropy balancing (step 1) and GLM (step 2). GLM refers to Generalized Linear Models and is specified with a gamma distribution and a log link function. All expenditure values are in US dollars. ‘Alternative’ refers to a specification in which we dropped information on forest activities and wealth status. Robust standard errors were used and are depicted in parentheses. */**/*** denote significance levels at 10/5/1 percent respectively.
