# Supplementary material for: The economic burden of loiasis: A comprehensive cost-of-illness analysis of regionally representative, individual-level data from rural Gabon
Source: PLoS One. 2026 Feb 23;21(2):e0340689. doi: 10.1371/journal.pone.0340689 (PMC12928485; doi:10.1371/journal.pone.0340689)
Supplement: S9 Table — (DOCX) [file pone.0340689.s009.docx]

**S9 Table. Impact of loiasis on health costs (controlling for chronic diseases)**

| **Variable** | **Observations** | **Estimates** |
| --- | --- | --- |
|  | (1) | (2) |
| Direct medical costs | 1231 | 4.69  (27.84) |
| Direct non-medical costs | 1231 | 9.15  (4.03)*** |
| Indirect costs | 1231 | 23.01  (13.19)* |

Notes: Estimates refer to marginal effects and are obtained from a two-step process involving entropy balancing (step 1) and GLM (step 2). GLM refers to Generalized Linear Models. All expenditure values are in US dollars. A binary variable related to the presence (=1) or absence (=0) of chronic diseases is added as a covariate in the specification. 38 respondents did not know whether they have a chronic disease or not. Hence 1,231 observations. Robust standard errors were used and are depicted in parentheses. */**/*** denote significance levels at 10/5/1 percent respectively.
