# Supplementary material for: The economic burden of loiasis: A comprehensive cost-of-illness analysis of regionally representative, individual-level data from rural Gabon
Source: PLoS One. 2026 Feb 23;21(2):e0340689. doi: 10.1371/journal.pone.0340689 (PMC12928485; doi:10.1371/journal.pone.0340689)
Supplement: S11 Table — (DOCX) [file pone.0340689.s011.docx]

**S11 Table. Impact of loiasis on health costs (by wealth group)**

| **Variable** | **Below median** | **Median and above** |
| --- | --- | --- |
|  | (1) | (2) |
| Direct medical costs | 43.74  (26.56)** | 23.53  (41.90) |
| Direct non-medical costs | -0.59  (3.16) | 26.34  (11.53)*** |
| Indirect costs | -4.66  (14.36) | 82.76  (32.79)*** |
| Observations | 628 | 641 |

Notes: Estimates refer to marginal effects and are obtained from a two-step process involving entropy balancing (step 1) and GLM (step 2). GLM refers to Generalized Linear Models. All expenditure values are in US dollars. Wealth index was dropped as a control variable in both specifications. Robust standard errors were used and are depicted in parentheses. */**/*** denote significance levels at 10/5/1 percent respectively.
