# Supplementary material for: The economic burden of loiasis: A comprehensive cost-of-illness analysis of regionally representative, individual-level data from rural Gabon
Source: PLoS One. 2026 Feb 23;21(2):e0340689. doi: 10.1371/journal.pone.0340689 (PMC12928485; doi:10.1371/journal.pone.0340689)
Supplement: S12 Table — (DOCX) [file pone.0340689.s012.docx]

**S12 Table. Impact of loiasis on health costs (by working status)**

| **Variable** | **Not working** | **Working** |
| --- | --- | --- |
|  | (1) | (2) |
| Direct medical costs | -81.85  (76.56) | -8.10  (18.02) |
| Direct non-medical costs | 2.24  (5.13) | 8.97  (4.16)*** |
| Indirect costs | 53.21  (30.30)** | 14.60  (12.54) |
| Observations | 364 | 905 |

Notes: Estimates refer to marginal effects and are obtained from a two-step process involving entropy balancing (step 1) and GLM (step 2). GLM refers to Generalized Linear Models. All expenditure values are in US dollars. Wealth index was dropped as a control variable in both specifications. Robust standard errors were used and are depicted in parentheses. */**/*** denote significance levels at 10/5/1 percent respectively.
