# Supplementary material for: The economic burden of loiasis: A comprehensive cost-of-illness analysis of regionally representative, individual-level data from rural Gabon
Source: PLoS One. 2026 Feb 23;21(2):e0340689. doi: 10.1371/journal.pone.0340689 (PMC12928485; doi:10.1371/journal.pone.0340689)
Supplement: S13 Text — (DOCX) [file pone.0340689.s013.docx]

**S13 Text. Adjustment related to health insurance**

During the survey, the respondents provided information on their out-of-pocket expenditures, i.e. the costs they incurred when they went to a hospital, to a traditional healer or to a given health center. However, given that 78 percent of our sample is covered by the public health insurance, namely the Caisse nationale d’assurance maladie et de garantie sociale (CNAMGS), that the insurance supports an important share of the total healthcare costs, and in order to gather information on the full cost of a patient, we complemented the self-reported expenditures with the CNAMGS share of costs.

First, we collected administrative data from the Albert Schweitzer Hospital in Lambaréné, Gabon, so that we could rely on real-world data to generate the adjustment rates. This allowed us to define the share of costs supported by the CNAMGS, depending on whether they were treated in inpatient or outpatient care, but regardless of the disease and disease severity. We obtained the following results: the CNAMGS covered 3.88 times the amount paid by the patients received in inpatient care, and 3.63 times the amount paid by the patients received in outpatient care. These figures are in line with the usual coverage rate of the CNAMGS in community health centers and hospitals, which is 80% of the healthcare costs.

We then added the insurance costs to the out-of-pocket expenses of all the patients who declared that they were covered by the public insurance, but only to the following cost categories: consultation, medicine and medical devices, diagnostic tests, and accommodation. Expenditures on transportation, informal payments, caregivers, and the monetary value of time spent (e.g. for travelling) were not included.
