# Supplementary material for: The economic burden of loiasis: A comprehensive cost-of-illness analysis of regionally representative, individual-level data from rural Gabon
Source: PLoS One. 2026 Feb 23;21(2):e0340689. doi: 10.1371/journal.pone.0340689 (PMC12928485; doi:10.1371/journal.pone.0340689)
Supplement: S14 Text — (DOCX) [file pone.0340689.s014.docx]

**S14 Text. Estimation of presenteeism**

We follow the Osterhaus’s method to measure days with reduced productivity caused by health issues. For that, we asked respondents about 1) the number of days they work although bothered by health problems, and 2) an evaluation of their performance at work from 0 (much worse than usual) to 10 (similar performance as usual) of those days. The productivity loss was determined by multiplying the number of days worked less efficiently by one minus the reported efficiency of those days. For instance, a hypothetical individual reported that he/she was bothered by health problems for two days. He/she declared a performance of five. The productivity loss will be: 2 * (1-0.5) = 1 workday. This lost workday will then be multiplied by the daily income to generate a monetary value of productivity loss for presenteeism.
