# Supplementary material for: The economic burden of loiasis: A comprehensive cost-of-illness analysis of regionally representative, individual-level data from rural Gabon
Source: PLoS One. 2026 Feb 23;21(2):e0340689. doi: 10.1371/journal.pone.0340689 (PMC12928485; doi:10.1371/journal.pone.0340689)
Supplement: S15 Text — (DOCX) [file pone.0340689.s015.docx]

**S15 Text. Estimation of average daily wage**

We estimate an average daily wage using the following strategy: during the interviews, we collected data on the monetary value of agricultural production and any sort of in-kind income (e.g., selling woods or herbs from the forest or own consumption of harvested agricultural products). In addition, we asked each household to report their financial income and any financial assistance (e.g., from family members) they received during the month prior to the interview. We then divided this total income (for each household) by the number of individuals who can participate in income generating activities in that household, i.e., individuals older than 10 years of age. We calculated the average of this parameter over the entire sample. This average parameter for all households was then divided by 24 (excluding the weekends), in order to obtain the daily wage. Finally, this daily wage was used to estimate the value of missed working days and reduced productivity.
