# Supplementary material for: The economic burden of loiasis: A comprehensive cost-of-illness analysis of regionally representative, individual-level data from rural Gabon
Source: PLoS One. 2026 Feb 23;21(2):e0340689. doi: 10.1371/journal.pone.0340689 (PMC12928485; doi:10.1371/journal.pone.0340689)
Supplement: S16 Text — (DOCX) [file pone.0340689.s016.docx]

**S16 Text. Entropy balancing and generalized linear model approach**

Entropy balancing is a preprocessing method widely used in the field of health economics. Its purpose is to achieve covariate balance in observational studies when the variable of interest is binary (i.e. with a treatment and a control groups). In this study, we estimated the weights of the control group vs. treatment group, for which the weight of one is assigned, on the following set of covariates: *Loa loa* infection (the variable of interest), age groups, gender, work in the forest, health status (namely a malaria infection), level of education and wealth index. Details on these variables are available in S1 Table. The weights for the estimation of the average treatment effect on the treated (ATT) are generated by solving the following (Ress & Wild, 2024):

$$\begin{matrix} w=\underset{w}{\mathrm{argmin}}\sum_{i:Z_{i}=0} w_{i}log\left( w_{i} \right)\text{ subject to } \\ \sum_{i:Z_{i}=0} w_{i}c_{j}\left( X_{i} \right)=\frac{1}{n_{1}}\sum_{i:Z_{i}=1} c_{j}\left( X_{i} \right)\text{ for a set of functions }c_{j},j\in\{1,\cdots,J\}\text{ and } \\ \sum_{i:Z_{i}=0} w_{i}=1,w_{i}\geq0,i=1,\ldots,n\text{ where }n_{1}\text{ is the number of subjects in the treated group. } \end{matrix}$$

The expected outcomes are identified by

$$E\left[ Y_{i}(0)\mid Z_{i}=1 \right]=\sum_{i:Z_{i}=0} w_{i}Y_{i}\text{ and }E\left[ Y_{i}(1)\mid Z_{i}=1 \right]=\sum_{i:Z_{i}=1} \frac{1}{n_{1}}Y_{i}.$$

Once we obtained the weights and following Hainmueller (2012) methodology, we performed a generalized linear regression of each health-related cost category separately (the direct medical, direct non-medical, and indirect costs) on the reweighted *Loa loa* variable and adding village fixed effects. We used a gamma distribution and a log link function. After that, we derived the estimated marginal effect of loiasis on healthcare costs.
