# Supplementary material for: The economic burden of loiasis: A comprehensive cost-of-illness analysis of regionally representative, individual-level data from rural Gabon
Source: PLoS One. 2026 Feb 23;21(2):e0340689. doi: 10.1371/journal.pone.0340689 (PMC12928485; doi:10.1371/journal.pone.0340689)
Supplement: S17 Text — (PDF) [file pone.0340689.s017.pdf]

STRUCTURE

<u>Sociodemographic</u>

A. Sociodemographic characteristics
B. Dwelling characteristics
C. Employment and income
D. Personality and social preferences
E. Cognitive ability

<u>Health</u>

F. General health assessment
G. Health insurance, attitudes towards medicine and clinical trials
H. Health consumption: Inpatient care
I. Health consumption: Outpatient care
J. Indirect costs

<u>*Loa loa*</u>

K. Raploa
L. Intervention
M. Questions regarding understanding the intervention and mechanism
N. Decision for *Loa loa* screening & clinical trial participation
O. Understanding decision-making

Legend

Orange-red: Notes for enumerators and programming;

<u>Instructions for enumerators</u>: If a person does not understand a question, try to explain or rephrase the question. If a person does not know the exact number, always ask them to provide an estimate. It is also possible to read out the response items in case respondents do not know how to answer a question. Response items should in general not be read out loud to start with, except if it is indicated in the instructions.

Blue: Coding: numbers for unique answers; letters for multiple answers; 99 Don't know/Refused to answer

---

Welcome!

Dear madam/sir,

my name is _____. I would like to invite you to our survey that is about health, especially a disease called loiasis. The aim of the survey is to measure the costs of this disease and to get to know more about your health behavior. It includes questions about your health and your personal background. It also includes a few small exercises.

For participating in this interview, we give you a small gift as phone credit worth 1,000 FCFA. During the exercises – which are voluntary – this amount might change eventually. During the interview we will explain more about these exercises.

The participation in this interview is voluntary. You can stop at any time. The information you will share with us if you participate in this study will be kept completely confidential to the full extent of the law.

Please read and listen carefully to all of the instructions. In total, the study takes around 1 hour.

Thank you very much for participating!

Declaration of consent

I have understood all the mentioned points above and I declare my consent to participate in this survey.

0 No 1 Yes

Only if the declaration of consent is agreed, the survey can start. If no consent is given, politely say goodbye.

SURVEY

Z1A Village: ______________________

Z2A Household ID: ___________________

Please insert the household ID given in the household booklet.

Z3A Individual ID: ____________________

Please insert the individual ID given in the household booklet.

Z3B First and last name of participant: _______________

Z3C Picture of the ID card: _______________

Z4A Enumerator name: _______________________

Z4B Enumerator ID: _______________________

A Socioeconomic characteristics

We would like to start the interview with some general questions.

A1 What is your sex? 0 Male 1 Female

A2 How old are you? __________ years old

A3 In which region of Gabon were you born?

1 Estuaire 2 Haut-Ogooué 3 Moyen-Ogooué 4 Ngounié 5 Nyanga 6 Ogooué-Ivindo

7 Ogooué-Lolo 8 Ogooué-Maritime 9 Woleu-Ntem 10 another country

A4 Which ethnic group(s) do you belong to?

A Akele B Eshira (Guisir) C Fang D Kota E Myene F Nzebi G Obamba H Punu I Teke

J Other:_____

A5 What is your religion?

1 Catholic 2 Protestant 3 Charismatic Christian 4 Muslim 5 Traditional beliefs
6 Other: ___________ 7 None 99 NA/DK

A6 What is your relationship to the household head?

1 Household head 2 Spouse (Father/mother (in law)) 3 Child 4 Sibling

5 Other relatives (e.g. uncle/aunt/cousin) 6 Grandfather/-mother 7 Grandchild
8 Other: _____________

A7 *If A6=1* How many persons live in the household? No:______________________

A8 *If A6=1* How many of those are children younger than 10 years? No:________________

A9 What is the highest level of education you completed?

0 No education 1 Primary school 2 Secondary school 3 Higher education

4 School of Life 5 Muslim school 6 Other_____?

B *If A6=1* **Dwelling characteristics (DHS)**

B2 Does your household have:

Please ask about functional devices.

B2.1	Electricity?	0 No	1 Yes	99 NA/DK
B2.2	A radio?	0 No	1 Yes	99 NA/DK
B2.3	A television?	0 No	1 Yes	99 NA/DK
B2.4	A décodeur	0 No	1 Yes	99 NA/DK
B2.5	A computer (desktop/laptop)?	0 No	1 Yes	99 NA/DK
B2.6	A refrigerator/freezer?	0 No	1 Yes	99 NA/DK

B3 Does any member of the household own:

B3.1	A watch?	0 No	1 Yes	99 NA/DK
B3.2	A mobile phone?	0 No	1 Yes	99 NA/DK
B3.3	A bicycle?	0 No	1 Yes	99 NA/DK
B3.4	A motorcycle or motor scooter?	0 No	1 Yes	99 NA/DK
B3.5	A car or truck?	0 No	1 Yes	99 NA/DK
B3.6	A boat with a motor?	0 No	1 Yes	99 NA/DK
B3.7	A boat without a motor?	0 No	1 Yes	99 NA/DK

B4 What is the main source of drinking water for members of your household?

1 Piped water

2 Tube well or borehole
3 Dug well
4 Water from spring/rainwater
5 Tanker truck/cart with small tank
6 Surface water (river/dam/lake/pond/stream/canal/irrigation channel)
7 Bottled water
8 Other: ____

B5 Compared to other people in your village, would you consider yourself and your household to be …

Read the answers out loud.

1 Very poor 2 poor 3 middleclass 4 rich 5 very rich

C Employment and income

C1 Which of the following situations applies best to you?

Read the answers out loud.

1 I am employed.
2 I am working in my own (small) business/shop.
3 I am working outside the forest on my farm/plantation
4 I am working in the forest (e.g. on plantations).
5 I am fishing.
6 I am a freelancer/casual worker.
7 I work as family member to help with things (e.g. in agriculture/household).
8 I am unemployed.
9 I am retired or cannot work due to health reasons
10 I am at school/apprentice.

Work in the forest includes plantation or farm activities in the forest as well as collecting wood or any similar activity; 3 includes farming activities on plants in the village or close by that are outside of the forest as well as more large-scale.

C2 *If C1<7* How would you classify the sector of your main occupation?

1 Agriculture
2 Fishery
3 Hunting in the forest/Gathering woods/plants/herbs in the forest
4 Public sector/armed forces
5 Administration, clerical
6 Professional/technical/managerial
7 Sales/Services
8 Manual work
9 Household and domestic
10 Priest/pastor/religious

C3 *if C1<7* How many hours have you worked in your main occupation in the last week? Please consider the previous week, i.e. from Monday to Sunday, for this and all the questions asking about "last week".

__________ hours

C4 If C1≠4 How many days did you spent working in the forest during the last month? Please consider the day you are doing the interview and then go backward. Please do the same for questions about "the last 3 months". ______________________________________days

C5 If C1≠4 How many hours have you worked in the forest in the last week?

__________ hours

C6 How many hours have you worked in total (taking all working activities together) last week?

____hours

C7 Besides the work and jobs that you have listed above: during the last twelve months, have you engaged in any other temporary work? 0 No 1 Yes

C7.1 *If C7=1:* For how long? _________A. month__________B. weeks__________C. days

If multiple jobs please add the combined number of months, weeks, and days.

C8 Now we would like to ask you a few questions regarding the income you make from the various activities. This includes cash, in-kind but also things that you might consume from your own work, e.g. from harvesting your own crops or animals.

C8.1 *If C1=1* Considering the work you did as employee in the last month: How much money did you earn in total in the last month? FCFA__________________________ 99 Don't know / NA

C8.2 *If C1=1* In this work did you, in addition, receive any in-kind benefits such as free food, refunded transportation costs, or items as reward? 0 No, 1 Yes

C8.3 *If C1=1 & C8.2=1* In this work how much were you paid in kind in the last month? Please try to roughly estimate the monetary value of this:
FCFA____

C9.1 *If C1=2 or 6* Excluding any work in agriculture/fishery/forestry/gathering: Considering the work you did as self-employed or freelancer in the last month: How much money did you earn in total in the last month? FCFA__________________________ 99 Don't know / NA

C9.2 *If C1=2 or 6* In this work did you, in addition, receive any in-kind benefits such as free food, refunded transportation costs, or items as reward? 0=No, 1= Yes

C9.3 *If C1=2 or 6* In this work how much did you receive in kind in the last month? Please try to roughly estimate the monetary value of this:
FCFA____

C10.1 *If A6=1* Considering your household (all members together): Could you tell us the number of crops you were growing over the last 12 months?

A maximum of five (5) crops is allowed. If you grew more, please describe <u>the five most important crops</u> on your farm below.

The question includes both crops that are sold and that are for own consumption

No.	Please name one/another crop that you are growing.<br><br>(Cocoa, coffee, maize, cassava, cassava leaves, aubergine, folon, wheat, rice, rubber, sugar cane, please specify if other)	How much of this crop did you harvest over the last 12 months?	Unit for harvest	If you were to sell a unit at the local market/trader how much would you receive for 1 unit?
1				
2				
3				
4				
5				

C10.2 *If A6=1* Again considering your household (all members together): Could you tell us the number of animals/fishes you were growing/fishing over the last 12 months?

A maximum of five (5) animals/fishes is allowed. If you grew more, please describe the five most important animals/fishes you were growing/fishing.

Please refer to animals that are either grown for your own household consumption or that you will sell on the market.

No.	Name of the animal<br><br>(Cattle, sheep, pigs, goats, chicken, fish, turkey, duck, please specify if other)	Quantity harvested	Unit for harvest	If you were to sell a unit at the local market/trader how much would you receive for 1 unit?
1				
2				
3				
4				
5				

C11 *If A6=1* Now we would like to ask you something about your household's forest activities. Beside what you mentioned to grow: How much money have you earned from selling things (animals/items/wood/herbs) in the last month?
FCFA_________________________________ 99 Don't know / NA

C12 *If A6=1 & C1=9* How much money did you receive from pensions in the last month?
FCFA__________________________________ 99 Don't know / NA

C13 *If A6=1* What is the total amount of money (cash and in-kind) that your household received/earned in the last month? Please try to roughly estimate it.
FCFA ___________________________________ 99 Don't know / NA

D Personality and social preferences

D1 Big 5 (BFI-10) (openness, agreeableness, conscientiousness, neuroticism)

In the following, I will read out statements concerning personal characteristics. Please tell me whether you disagree, disagree a little, neither agree nor disagree, agree a little or strongly agree to any statement.

Give the participant the scale with the answering options.

	I see myself as someone who…	Disagree strongly (1)	Disagree a little (2)	Neither agree nor disagree (3)	Agree a little (4)	Agree strongly (5)
D1.1	is generally trusting.					
D1.2	tends to be lazy.					
D1.3	is relaxed, handles stress well.					
D1.4	is outgoing, sociable.					
D1.5	tends to find fault with others.					
D1.6	does a thorough job.					
D1.7	gets nervous easily.					
D1.8	has an active imagination.					
D1.9	is very religious.					

D2 Social Desirability-Gamma Short Scale + *Personal optimism scale (Gavrilov-Jerkovic et al. 2014)*

		Disagree strongly (1)	Disagree a little (2)	Neither agree nor disagree (3)	Agree a little (4)	Agree strongly (5)
D2.1	In an argument, I always remain objective and stick to the facts.					
D2.2	Even if I am feeling stressed, I am always friendly and polite to others.					
D2.3	When talking to someone, I always listen carefully to what the other person says.					
D2.4	I am facing my future in an optimistic way.					
D2.5	I worry about my future.					
D2.6	It often seems to me that everything is gloomy.					

D3 Global preference survey (risk, time preference and altruism, positive reciprocity, trust)

D3 In the following questions, we will use another scale (please show the scale card) that goes from 0 to 10. You can also use any number between 0 and 10 to indicate where you fall on the scale, using 0, 1, 2, 3, 4, 5, 6, 7, 8, 9, or 10, where 0 means you are completely unwilling to do something and 10 willing to do something.

Please tell me, in general, how willing or unwilling you are to take risks, using that scale from 0 to 10.

Completely unwilling to take risks 0 1 2 3 4 5 6 7 8 9 10 Very willing to take risks

99 Don't know

D4 I will ask you some further questions. Please answer them all on the scale from 0 to 10:

How willing are you to give to good causes without expecting anything in return?

Completely unwilling to do so 0 1 2 3 4 5 6 7 8 9 10 Very willing to do so

99 Don't know

D5 When someone does me a favor, I am willing to return it.

Completely unwilling to do so 0 1 2 3 4 5 6 7 8 9 10 Very willing to do so

99 Don't know

D6 How willing are you to give up something that is beneficial for you today in order to benefit more from that in the future?

Completely unwilling to do so 0 1 2 3 4 5 6 7 8 9 10 Very willing to do so

99 Don't know

D7 Please tell me, if in general you are an impatient person, or someone who always shows great patience using a scale from 0 to 10, where 0 means you are "very impatient" and 10 means you are "very patient."

Very impatient - 0 1 2 3 4 5 6 7 8 9 10 – very patient

99 Don't know

D8 Please think about what you would do in the following situation. You are in an area you are not familiar with, and you realize that you lost your way. You ask a stranger for directions. The stranger offers to take you to your destination. Helping you costs the stranger about 2000 FCFA in total. However, the stranger says he or she does not want any money from you. You have six presents with you. The cheapest present costs 500 FCFA, the most expensive one costs 2500 FCFA. Do you give one of the presents to the stranger as a "thank you" gift?

1 No, would not give present

(If Yes, ask:) Which present do you give to the stranger?

2 The present worth 500 FCFA

3 The present worth 1000 FCFA

4 The present worth 1500 FCFA

5 The present worth 2000 FCFA

6 The present worth 2500 FCFA

99 (DK/NA)

D9 Imagine the following situation: Today you unexpectedly received 800 FCFA. How much of this amount would you donate to a good cause? (Values between 0 and 800 are allowed)

________________________ 99 Don't know/NA

D10 I do not understand why some people spend their lifetime fighting for a cause which they do not benefit from directly.

Completely disagree with statement 0 1 2 3 4 5 6 7 8 9 10 Completely agree with statement

Trust based on Afrobarometer

D11 Generally speaking, would you say that most people can be trusted or that you must be very careful in dealing with people?

1 Must be very careful 2 Most can be trusted 99 Don't know

D12 How much do you trust each of the following, or haven't you heard enough about them to say?

	Not at all 1	Just a little 2	Somewhat 3	A lot 4	Have not heard enough / NA 99
D12.1 Family members					
D12.2 Neighbors					
D12.3 CERMEL					
D12.4 (Conventional) Medicine					
D12.5 Traditional healers					
D12.6 Nurses/health workers of CERMEL					
D12.7 Medical doctors of CERMEL					

D13 **Experimental exercises**

D13.1 **Trust exercise**

Now we will start a small exercise.

In this exercise, you can gain or lose phone credit. The exercise involves an interaction with a nurse or a field worker from CERMEL. We recorded the decision of the person you interact with prior to this session. You should know that neither you nor the person from CERMEL knows about the identity of the other. Meaning, we will not tell you the name of that person. Likewise, that person will not receive your name and address either.

Let me start with explaining the task. **We will provide you with another 500 FCFA of phone credit**. Note that you now would get 1,500 FCFA of phone credit from us (1,000 FCFA for participating in the survey and 500 FCFA now).(Show the phone credit to the participant). This will be yours. **You can send that phone credit to a randomly chosen nurse or field worker from CERMEL**. You can decide to send all the 1,500 FCFA but you can as well decide to send

nothing (0 FCFA) or just a fraction of the 1,500 FCFA. If you send a fraction, we would like to ask you to specify the amount in multiple of 500, e.g. 500 or 1,000 or 1,500 FCFA.

Now there is an important aspect I should mention. **The person that you send credit to can send it back to you**. This, however, will be completely up to that person and is not up to you and not up to us either. We explained to that other person the rules of the interaction as we do now to you. Therefore, the person knows that it was up to you whether to send credit or not and what your bonus was. The nurse or field worker from CERMEL decides as you will do whether he/she will send you back all of the credit, nothing or a fraction in multiple of 500 FCFA. **If the other person decides to give you credit back, we will triple that amount**. This means that you will receive three times the credit that the person from CERMEL will send you back in case you send something to that person. In your best case, you get 3 times the amount that you sent to him/her back. In the worst case he/she sends you nothing back and you will lose all the credit you have sent. Do you understand?

Let me give you an **example:** Imagine you would send 1,000 from the 1,500 FCFA to the person from CERMEL. Then the person decides whether he/she will return you something. Let's say the person returns to you 500 FCFA. Then this amount would be tripled, and you would receive from us 500 * 3 = 1,500 FCFA, while the person from CERMEL receives 500 FCFA. So in total you would gain 2,000 FCFA, i.e. the 1,500 FCFA that you have received back and the 500 FCFA from the beginning that you have kept. Do you understand?

Before I continue to the interaction session, I would like to quickly check whether you understood the rules I have just explained. In case there is some misunderstanding I am happy to explain it again. I will ask you some understanding questions. May I?

1) What is the maximum amount you can send to the person from CERMEL? *

2) What is the minimum amount you can send to the person from CERMEL? *

3) What is the maximum amount you could pocket for yourself in this interaction? *

understanding	This is an overview of the given and correct answers. Please explain again, where necessary.<br>1) What is the maximum amount you can send to this other person?<br>His/her answer: ${trustg_q1};<br>correct answer: 1,500 FCFA<br>2) What is the minimum amount that you can send to this other person?<br>His/her answer: ${trustg_q2};<br>correct answer: 0<br>3) What is the maximum amount you could pocket for yourself in this interaction?<br>His/her answer: ${trustg_q5};<br>correct answer: 4,500 FCFA

Are you fine? Shall I explain some aspects of the interaction again?

Please give the respondent time to think about their decisions. Do not rush the respondent. If respondent likes to have more time, please give it to him/her.

Let's start the real exercise. Here is a phone credit of 1,500 FCFA. From that credit, do you want to send something to the nurse or the field worker from CERMEL? You can send nothing, all or a fraction which is a multiple of 500 FCFA. We recorded the responses of the other person already and depending on your decision, that person might have sent credit back to you or not.

The interaction remains anonymous. I will let you know afterwards what the person from CERMEL decided, and you will receive three times the amount returned.

Now I would like to ask you to make your decision. How much of the 1,500 FCFA would you like to send to that person? Please take your time to think about your decisions. There is real phone credit at stake here. The decision on how much you want to send is completely up to you. Again, you can send nothing or everything, or just a part of it.

Amount sent: ___________________________________

Program that the answer will be given randomly matched to one of the answers the nurses and field workers have given.

Okay, you have sent [amount sent] FCFA to the person from CERMEL. The person has decided to send you x FCFA back. We will multiply this amount by 3, i.e. 3 * x FCFA. This means that you will receive, in the end, x FCFA from us: 3*x plus the amount you have kept (1,500 – amount sent) FCFA.

If the respondent did not send money to CERMEL employee: As you did not send money to the CERMEL employee, you will keep the amount of 1,500 FCFA.

D13.2 **Donation game**

We would like to give you the chance to donate some of your earned money today. Again, this is completely voluntary and up to you. It is perfectly fine not to give anything. This money was for the interview and your participation in the exercise.

But if you are interested, please let me explain the following to you. We work together with an NGO called "Association la Maison d'Hermann". The NGO provides food, school supplies, and other necessary materials to poor Gabonese children. Would you perhaps be interested in donating to the NGO? If yes, how much? Again, it is completely fine to donate nothing too. This is just a minor topic and we thought we just ask you.

E Cognitive ability

E1 Can you read Latin numbers and letters?

0 I cannot read numbers and letters. 1 I can read letters but not numbers. 2 I can read numbers but not letters 3 I can read both.

Skip section E if person cannot read numbers and letters!

E2 If E1=3 or E1==2 ***Digit span test***

I will show you now a sequence of numbers, which appear on the screen. Please remember the numbers in the order they appear.

Sequence of randomly shown numbers.

Please give the person not more than 1-minute time to remember the numbers. It is important that the numbers are only shown once.

E2.1 Please tell me the numbers in the same order as they have appeared:

Number: ______________________________

E2.2 Please recall the numbers now in reverse order (backwards):

Number reverse: ______________________________

E3 If E1=3 or E1==1 ***Reading skills***

E3.1 Now I would like you to read this sentence to me.

Show a sentence

To be coded by the enumerators

1 Person cannot read at all. 2 Person can read part of the sentence. 3 Person can read the full sentence. 4 Person has vision problems/refused to read.

E4 If E1=3 or E1==2 ***Analytical thinking (IFLS)***

Now we will show you several series of numbers on the computer screen. In each series, there will be one number that is missing. The missing number will be indicated by a question mark "?". Please look at the pattern of the numbers. Based on this pattern, tell me what is the number that is missing. For example, if you see the following, what number should go in the question mark?

The numbers shall be shown to the person for not more than 1 minute. If respondent answers incorrectly or did not answer, then say: The answer we were looking for is 6.

3	4	5	?

Do you understand the directions for this task?

For respondents with 0 correct answers:

I will present you three further sequences. Please tell me what is the missing number based on the pattern.

E4.1	1	2	3	?
E4.2	6	5	4	?
E4.3	12	?	16	18

For respondents with 1 correct answer:

I will present you three further sequences. Please tell me what is the missing number based on the pattern.

E4.1	5	?	3	2
E4.2	4	7	10	?
E4.3	?	4	6	8

F General health assessment

The following questions deal with your health status.

F1 In general, would you say your health is:

Read the answers out loud.

1 poor 2 fair 3 good 4 very good 5 excellent

F2 Do you have any disability or handicap?

0 No 1 Yes

F2.1 *If F2=1* What kind of disability do you have?

I have a problem with ….

A vision B mobility C hearing D cognitive E other:______

F.2.2 *If F2=1* Is the problem very severe, severe, moderate, mild or very mild?

1 very severe 2 severe 3 moderate 4 mild 5 very mild

F3 During the past 4 weeks, have you suffered from an acute illness or injury?

0 No 1 Yes

F4 *If F.3=yes*: Which one(s)? _____________________________ 99 Don't know / NA

F5 *If F.4=99*: Please describe briefly the symptoms.

____

F6 Do you regularly use/sleep under any mosquito nets?

0 No 1 Yes

F7 Do you use any insect repellents regularly?

0 No, not at all 1 Yes, sometimes 2 Yes, most of the times

F8 *If A1=1* Are you currently pregnant?

0 No 1 Yes 99 Don't know / NA

F9 Do you know whether you have any (other) disease or illness? 0 No 1 Yes 99 Don't know / NA

F10 *If F9=yes*: Which ones?

A Pneumonia B heart disease C poliomyelitis D diarrhea E measles F rubella G mumps
H hepatitis I AIDS J dengue fever K malaria L hypertension M chronic belly ache
N chronic headache O cancer P lung disease Q kidney disease R Other:____________

F11 **PHQ-4 ITEM LIST (DEPRESSION + ANXIETY)**

Over the last two weeks, how often have you been bothered by the following problems?

		Not at all 0	Several days 1	More than half of the days 2	Nearly every day 3
F11.1	Feeling nervous, anxious or on edge				
F11.2	Not being able to stop or control worrying				
F11.3	Feeling down, depressed or hopeless				
F11.4	Little interest or pleasure in doing things				

G Health insurance, attitudes towards medicine and clinical trials

G1 Are you covered by any type of health insurance (e.g. CNAMGS)?
0 No 1 Yes

G2 *If G.1=yes:* Is it a private or public insurance? 1 Public 2 Private

G3 *If G.1=yes*: Does the insurance cover treatments of worm infections?

0 No 1 Yes 99 Don't know / NA

G4 *If G.1= 1 & G.2=1 & C1=1 or G.1=yes, G.2 = 2*: How much do you typically pay for the health insurance per month?

FCFA_______________ a month 99 Don't know / NA

G5 Which health care service system do you generally prefer more: traditional or modern medicine?

0 Traditional 1 Modern 2 Both

G6 Please tell me whether you "strongly disagree", "disagree", "neither agree nor disagree", "agree", or "strongly agree" to the following statements.

		Strongly disagree 1	Disagree a little 2	Neither agree nor disagree 3	Agree a little 4	Strongly agree 5

G6.1	Modern medicine has cures for most diseases.					
G6.2	Modern medicines can do as much harm as good.					
G6.3	I would like to go to medical checkups but I cannot afford it.					
G6.4	I avoid medical checkups because they make me uncomfortable.					
G6.5	I take good care of my health.					

G7 Do you know what a clinical trial is? 0 No 1 Yes

If no: A clinical trial is a research study in which a new medical treatment, for instance a new drug, gets tested. In such a study, some persons will try out the new drug and others will receive a placebo or the standard treatment available. To evaluate the effects of the new drug, the health changes and behavior of the persons will be monitored and compared.

G8 Have you ever participated in a clinical trial? 0 No 1 Yes

G9 Please tell me in your opinion how trustworthy are clinical trials on the following scale.

Very untrustworthy 0 1 2 3 4 5 6 7 8 9 10 very trustworthy

G10 ***Ohmann and Deimling: General attitudes towards clinical trials***

In your opinion, what effects does a clinical trial have…

scientific progress? 0 No 1Yes
improvement of clinical trial patient's health? 0 No 1Yes
improvement of other people's health? 0 No 1 Yes

Serving the interest of the pharmaceutical industry? 0 No 1 Yes

Other: ______________________________________

G11 *If G8=0:* Generally speaking, can you imagine participating in a clinical trial?

0 No 1 Yes 99 Don't know

G12 *If G8=1:* Can you imagine participating in a clinical trial again?

0 No 1 Yes 99 Don't know

G13 How many people of your village do you expect to participate, in general, in a clinical trial in case they were asked?

0 Almost no one 1 some 2 around half 3 many 4 almost all 99 Don't know/ NA

Health care consumption: **consumption and costs for health service**

In the following section, we are interested in your health care consumption. Please answer the questions for the time period of the last three months.

H Health consumption: Inpatient care

H1 Have you been in inpatient care (staying overnight) for health-related reasons in the last 3 months? 0 No 1 Yes

If H1=no:

H1.2*:* In the last 3 months did you refrain from any inpatient care although it would have been necessary due to economic reasons?
0 No 1 Yes

If H1.2=yes:

H1.3 How often was this the case? No: ____________________

Then continue with next section -

H2 In which health care facility did you receive inpatient care in the last 3 months?

If response item is not named by the respondent, ask explicitly

1 Private hospital 2 Public hospital 3 Private health center
4 Public health center 5 Other: _________________

H3 - Please ask the following question for each facility mentioned in H2! –

Have you been in __(answer H2)___ multiple times in the last three months?
0 No 1 Yes

H3.1 *If H3=yes:* How many times?

Number: ________________________

H3.2 In the last 3 months how many days did you spend in total (all your stays together) in this health care facility?

________________________________ days

H3.3 What was the reason for your stay? _______________________________

The following questions relate to your last stay.

H4 *If multiple answers are given in H2:* In which facility did you stay last?

A Private hospital B Public hospital C Private health center
D Public health center E Other: _________________

H5 Did a caregiver or anyone else accompany you to this health care facility?
0 No 1 Yes

H5.1 *If H5=yes:* How many adult persons did accompany you?

____

H6 How much did you pay for show up fees / registration fees / consultation fees /examination?

FCFA____ 99 Don't know / NA

H7 Did you pay for any medication during or following your stay (e.g. pain killers, antibiotics, vitamins, etc.)? 0 No 1 Yes

H7.1 *If H7=yes:* How much?

FCFA____ 99 Don't know / NA

H8 Did you pay for any diagnostic tests before, during or following your stay (e.g. blood test, stool test, urine test, etc.)? 0 No 1 Yes

H8.1 *If H8=yes:* How much?

FCFA____ 99 Don't know / NA

H9 Did you pay for the purchase of any medical devices during or following your stay (e.g., blood pressure monitor, blood glucose monitor, walker, wheelchair, raised toilet seat, protective underwear, shower rails)? 0 No 1 Yes

H9.1 *If H9=yes:* how much?

FCFA____ 99 Don't know / NA

H10 Did you pay for any additional non-medical services during or following your stay (e.g., insurance forms, sending photocopies, doctor's certificate)? 0 No 1 Yes

H10.1 *If H10=yes:* How much?

FCFA____ 99 Don't know / NA

H11 Did you spend any <u>additional</u> money (on items not mentioned before, e.g. excluding examination fees) e.g. as tips for your own willingness or against your willingness which consider as an informal payment?

0 No 1 Yes

H11.1 *If H11=yes:* How much?

FCFA_______________________________________ 99 Don't know / NA

H12 How much did you pay for transportation?

FCFA_______________________________________ 99 Don't know / NA

H13 How much did you pay for accommodation?

FCFA_______________________________________ 99 Don't know / NA

I Health consumption: Outpatient care

I1 Read out loud all response possibilities.

Have you experienced the following conditions in the last 3 months?	*If yes:* How often have you	*If yes:* How much did you	*If yes:* How much did you	*If yes:* How much did you

		experienced this in the last 3 months? (Reponses: everyday, most of the days, a few days, once a week)	spend for medication?	spend for consultation?	spend for transportation?
Joint pain	0 No 1 Yes				
Muscle pain	0 No 1 Yes				
Transient joint swelling	0 No 1 Yes				
Itch	0 No 1 Yes				
headaches	0 No 1 Yes				
Problems with vision or infection of the eye	0 No 1 Yes				

Now we would like to ask you something about health care you have looked for in the last 3 months. This includes consultations or visits to formal and traditional doctors and nurses and pharmacies/vendors of medicine. Please only think of those where you did not stay overnight.

I2 Have you searched for such services in the last three months (other than just mentioned)?

0 No 1 Yes

If I2=no:

I2.1 Did you refrain from any consultation although it would be necessary out of economic reasons? 0 No 1 Yes

If I2.1=yes:

I2.2 How often was this the case? No: ____________________

Then continue with next section

I3

Have you visited the following health care facilities in the last 3 months?		*If yes:* How often have you visited this facility in the last 3 months?	*If yes:* What was the reason for your visit?
Private hospital	0 No 1 Yes		
Public hospital	0 No 1 Yes		
Private health center	0 No 1 Yes		
Public health center	0 No 1 Yes		
Doctor’s practice	0 No 1 Yes		
Traditional practice (healer)	0 No 1 Yes		
Pharmacy	0 No 1 Yes		

Any other health care service provider: ________________	0 No 1 Yes		

Please answer the following questions with respect to your last consultation (excluding pharmacy).

I4 How much time did you spend in total (for travelling, waiting and the consultation) for your last consultation?

Hour(s): Minutes:

I5 Did a caregiver or anyone else accompany you to your consultations at this health care facility? 0 No 1 Yes

I5.1 *If I5=yes*: How many adult persons did accompany you?

____

I6 How much did you pay for show up fees / registration fees / consultation fees /examination?

FCFA__________________________________ 99 Don't know / NA

I7 Did you pay for any medication during or following your consultation (e.g. pain killers, antibiotics, vitamins, etc.)? 0 No 1 Yes

I7.1 *If I7=yes*: How much? FCFA__________________________________

99 Don't know / NA

I8 Did you pay for any diagnostic tests before, during or following your consultation (e.g. blood test, stool test, urine test, etc.)? 0 No 1 Yes

I8.1 *If I8=yes*: How much? FCFA__________________________________

99 Don't know / NA

I9 Did you pay for the purchase of any medical devices during or following your consultation (e.g., blood pressure monitor, blood glucose monitor, walker, wheelchair, raised toilet seat, protective underwear, shower rails)? 0 No 1 Yes

J9.1 *If I9=yes*: How much? FCFA__________________________________

99 Don't know / NA

I10 Did you pay for any additional non-medical services during or following your consultation (e.g., insurance forms, sending photocopies, doctor's certificate)? 0 No 1 Yes

I10.1 *If I10=yes*: How much? FCFA__________________________________

99 Don't know / NA

I11 Did you spend any additional money as tips for your own willingness or against your willingness which consider as an informal payment (pls exclude those that were mentioned above already (e.g. consultation fees))? 0 No 1 Yes

I11.1 *If I11=yes*: How much? FCFA_________________________________

99 Don't know / NA

I12 How much did you pay for transportation?

FCFA___________________________________ 99 Don't know / NA

I13 Besides the cost for medication you already listed above, did you purchase additional medication (pain killers, antibiotics etc.), e.g. at a pharmacy, in the last month?

0 No 1 Yes

I13.1 *If I13=yes*: How much did you pay in total for medication?

FCFA_______________________________ 99 Don't know / NA

J Indirect costs: Short-form Health and Labor questionnaire (SF-HLQ)

J1 Did health problems oblige you to be off work at any time in the past month?

0 No

1 Yes, I missed …………days……. hours of work

- If no continue with question J6 -

J2 Could you not work for a period longer than the past month because of health problems?

0 No

1 Yes, I was ill for (J2.1)_________days_________weeks_____months

J3 *If J1 = yes:* Please estimate how much income (if at all) you lost due to health-related absence from work.

FCFA __________________________________ 99 Don't know / NA

J4 During illness, did anybody take care of you? 0 No 1 Yes

J5 *If J4=yes:* Did this person / these persons miss work due to the fact that they were taking care of you?

0 No 1 Yes

J5.1 *If J5=yes*: How long was the person / were the persons absent from work?
_______hours_______days____weeks

J6 Was your own job performance adversely affected by health problems during the past month?

0 No, not at all

1 Yes, slightly

2 Yes, very much

J7 *If J6=yes*: On how many days during the past month did you perform work, although you were bothered by health problems?

___________________ days

J8 *If J6=yes*: Please rate how well you performed on the days you went to work even though you were bothered by health problems. 0 indicates a much worse performance than usual and 10 that your work was not affected. (For instance, you had concentration problems, needed to work at a slower pace, or postpone work.)

0 1 2 3 4 5 6 7 8 9 10

J9 Did other people take over and perform your usual (household) tasks in the past month because of your health problems?

0 No

1 Yes, namely (more than one answer is possible):

J9.1 family members for.................... hours

J9.2 other persons receiving no pay for.................... hours

J9.3 paid care for.................... hours

J10 *If F3=yes:* You have mentioned before that you have suffered from an injury / acute illness in recent time: How did you cope (economically) with it? Possible themes refer to your job or work in agriculture/forestry or how other household members helped.

Read out loud the categories that are underlined

1 No problems to cope with the injury/illness
Adjust working time
A Took up an additional occupation: agricultural wage employment
B Took up an additional occupation: opened business
C Took up an additional occupation: non-farm wage employment
Adjust farming
D Worked more time on the farm
E Diversify agricultural portfolio
F Substitute crops
G Reduced production inputs
H Sold livestock, land, storage, other assets
Adjustments related to household
I Took children out of school
J Sent children to relatives/friends
K Adult migrated to look for job
L Adult migrated to live with relatives/friends
M Adult migrated to marry
N Other: _________________________________

J11 *If F3=yes:* Did you have to reduce your household consumption due to that?

0 No, not at all 1 Yes, somewhat 2 Yes, a lot

K Raploa

Next, we would like to talk with you about *Loa loa* filariasis (or „les filaires“).

K1 Have you heard of *Loa loa* filariasis (or „les filaires“) before?

0 No 1 Yes 99 Don’t know / NA

If K1=0 OR K1=99 explain: Loa loa filariasis is a skin and eye disease caused by the nematode worm Loa loa, contracted by bites of a deer fly or mango fly, the vectors for Loa loa. The worm migrates throughout the body of humans, occasionally crossing into tissues of the eye where it can be easily observed. Loa loa can be painful when moving about the eyeball or across the bridge of the nose. The disease can cause red itchy swellings below the skin called "Calabar swellings", joint and muscle pain. The symptoms often appear not until half a year after the bite. Hence, also persons without symptoms can be infected. We will tell you more about Loa loa later.

K2 Have you ever experienced or noticed worms moving along the white part of your eye?

0 No 1 Yes 99 Don't know / NA

K3 Have you ever had the condition in this picture?

0 No 1 Yes 99 Don't know / NA

K4 *If K2=1 or K3 = 1:* The last time you had this condition, how long did the worm stay before disappearing?

___________ days

K5 *If K2=1 or K3 = 1:* How often have you experienced this in the last three months?

____

K5.2 *If K5=0:* Have you experienced this in the last twelve (12) months?

0 No 1 Yes

K6 Have you ever been screened or tested for *Loa loa* (or „les filaires")?

0 No 1 Yes

K7.1 How likely do you think it is that you are currently infected with *Loa loa* filariasis (or „les filaires")?

1 No, highly unlikely 2 No, rather unlikely 3 Yes, rather likely 4 Yes, highly likely

K7.2 Assuming that you are infected with *Loa loa*, how much would you be willing to pay for a treatment of *Loa loa* (or „les filaires")?

FCFA_________________________

K8 Have you ever been treated for *Loa loa* (or „les filaires")? 0 No 1 Yes

K9 *If K8=1*

Where have you been treated?	How much did you pay for the treatment (consultation fees, diagnostic tests, etc.)?	How much did you pay for medication and additional medical devices?	How much did you pay for accommodation and travel?
Traditional healer			
Doctor's practice			

Health center / hospital			
Pharmacy			

K10 Have you ever been treated for any other worm infection? 0 No 1 Yes

K11 ***Social norms & Personal Attitudes towards Loa loa and medical screening***

Please tell us to what extent you agree or disagree with the following statements:

		Disagree 1	Disagree a little 2	Neither agree nor disagree 3	Agree a little 4	Agree 5
	Descriptive norms scale					
K11.1	In my village few people look for medical screening to see whether they have *Loa loa*.					
	Injunctive norm scale					
K11.2	The majority of people in my village care about whether they have *Loa loa* or not.					
	Outcome expectations scale					
K11.3	In my village many people think that it is too expensive or time consuming to get screened for *Loa loa*.					
	Behavior publicness scale					
K11.4	It is easy to see whether a person of my village has *Loa loa* or not.					
K11.5	*Loa loa* is a serious health issue.					
K11.6	Knowing through a medical test whether I have *Loa loa* would help me treat *Loa loa*.					
K11.7	Having *Loa loa* does not affect my daily living much.					

L Intervention

Please look at the household list/booklet which intervention you should provide. Overall, section L shall last for a maximum of 10 minutes. The enumerator shall self-fill out section L once the intervention was conducted.

L1 Which version of the intervention did you conduct with this person?

1 Version A: Control 2 Version B: Altruism 3 Version C: Trust

L2 On a scale from 0 to 10, how well do you think the respondent understood your intervention?

0 1 2 3 4 5 6 7 8 9 10

M Questions regarding understanding the intervention and mechanism

M1 ***Discrete emotions questionnaire***

Imagine you would get tested for *Loa loa* and that you would then receive a test result. To what extent do you expect experiencing following emotions?

		Not at all 1	Somewhat 2	Moderately 3	Quite a bit 4	Very much 5
M1.1	Calm					
M1.2	Mad					
M1.3	Sad					
M1.4	Worry					
M1.5	Happy					

M2 Imagine you would receive a positive test result and that you would participate in the *Loa loa* clinical trial. To what extent do you expect experiencing the following emotions during the trial participation?

		Not at all 1	Somewhat 2	Moderately 3	Quite a bit 4	Very much 5
M2.1	Calm					
M2.2	Mad					
M2.3	Sad					
M2.4	Worry					
M2.5	Happy					

M3 ***Knowledge about Loa loa, screening and clinical trials***

We now would like to ask you a few questions about some of the topics we just talked about. Please indicate whether you think this is right or wrong.

		False 0	Correct 1	Don't know /NA 99
M3.1	Symptoms of *Loa loa* can be high blood pressure and heart problems.			
M3.2	*Loa loa* symptoms can be calabar swelling.			
M3.3	I will get paid for getting screened for *Loa loa*.			
M3.4	As part of the *Loa loa* clinical trial, participants have to take several new medications.			
M3.5	If I consent to the blood test for *Loa loa* screening, I would have to participate in the *Loa loa* clinical trial.			

M4 In the following section, I will read out statements about opinions related to *Loa loa* and the *Loa loa* clinical trial. Please tell me whether you disagree, disagree a little, neither agree nor disagree, agree a little or strongly agree to any of the statements.

		Disagree 1	Disagree a little 2	Neither agree nor disagree 3	Agree a little 4	Agree 5
M4.1	Participating in clinical trials can help many other persons.					
M4.2	I am a person who is generally willing to share with others without expecting something in return.					
M4.3	In case I am loiasis positive, I expect to be healthy after the clinical trial.					
M4.4	I worry about the confidentiality of my data and blood samples.					
M4.5	I hope that the children in our household will get good and cheap health care from CERMEL when I participate in the clinical trial.					
M4.6	*Loa loa* is a minor health issue.					

M5 Please tell me what is your opinion about the *Loa loa* medical screening on the following scales.

Very untrustworthy 0 1 2 3 4 5 6 7 8 9 10 very trustworthy

M6 Please tell me what is your opinion about the *Loa loa* clinical trial on the same scale.

Very untrustworthy 0 1 2 3 4 5 6 7 8 9 10 very trustworthy

N Decision for *Loa loa* screening & clinical trial participation

Before, we informed you about *Loa loa*, the screening for it and the *Loa loa* clinical trial. We now would like to ask you whether you would like to be tested for *Loa loa*. The test results should be available 1-2 weeks afterwards. This test is entirely voluntary. It is absolutely fine for us if you do not want to get tested.

N1 Would you like to get tested for *Loa loa*?

0 No 1 Yes 99 Don't know / NA

N2 In case you are diagnosed for *Loa loa*, is it likely that you would participate in the *Loa loa* clinical trial? The participation is voluntary. It is fine for us to be tested and not to participate in the clinical trial.

1 No, highly unlikely 2 No, rather unlikely 3 Yes, rather likely 4 Yes, highly likely

O Understanding decision-making

O1.1 If N1=1. DO NOT read out loud the answers.

What was the reason for your decision to participate in the *Loa loa* screening?

1 I want to know more about my health status

2 For my own health benefit

3 Possibility to get gratis *Loa loa* treatment

4 Possibility to talk to a doctor

5 For the benefit of the community

6 I felt obligated to do so

7 To help the researchers

8 To reassure that I am healthy

9 To have health care for my children

10 Other: ____________________________

O1.2 If N1=0. DO NOT read out loud the answers.

What was the reason for your decision not to participate in the *Loa loa* screening?

1 I already know my *Loa loa* status

2 I mistrust the doctors

3 I am afraid of the diagnosis

4 I am afraid of needles

5 I am sure that I am healthy

6 I felt obligated to do so

7 It would upset my family or friends

8 Other: ____________________________

O2 ***List experiment*** (50:50 randomization of List A and List B)

Please tell us how many of the following statements you agree with. There is no need to tell us which of these statements you agree with. Just tell us how many, e.g. 0, 1, 2, or 3.

O2.1 LIST A: I am now going to read to you 4 statements. How many of these 4 statements do you agree with?

Read out loud all statements.

1: I have been to Libreville.

2: I have more than one sister.

3: I felt pressured to get tested for *Loa loa*.

4: I consider myself to be a sports fan.

No. of statements you agree with: _______________

O2.2 LIST B: I am now going to read to you 3 statements. How many of these 3 statements do you agree with?

Read out loud all statements.

1: I have been to Libreville.

2: I have more than one sister.

3: I consider myself to be a sports fan.

No. of statements you agree with: _______________

O3.1 If N1=1 When deciding for yourself to get screened and possibly participate in the clinical trial, did you think about what other people in your family or in the village might have decided?

1No, not at all 2 No, not a lot 3 Yes, a bit 4 Yes, very much

O3.2 If N1=0 When deciding for yourself to not get screened and possibly participate in the clinical trial, did you think about what other people in your family or in the village might have decided?

1No, not at all 2 No, not a lot 3 Yes, a bit 4 Yes, very much

O4 What do you think – how many persons in this village (older than 18) will participate in the *Loa loa* screening?

1 Almost everyone 2 A majority but not most of the people 3 About half of all people
4 Less than half of the people but some 5 Very few or no one

O5 What do you think – how many people in this village (older than 18) will want to participate in the *Loa loa* clinical trial?

1 Almost everyone 2 A majority but not most of the people 3 About half of all people
4 Less than half of the people but some 5 Very few or no one

O6 Imagine you or a member of your household has a severe health issue. To which other households in this village would you talk to and ask for advice? Please mention not more than three.

1. _______________

2._______________

3._______________

O7 If N2=3 or 4 On a scale from 0 (highly unlikely) to 10 (very likely), how likely is it that you will discuss your participation in the clinical trial with other households in this village?

Highly unlikely 0 1 2 3 4 5 6 7 8 9 10 very likely

O8 *If A6=1* Has any member of your household ever received health care at CERMEL?

0 No 1 Yes 99 NA/DK

O9 *If A6=1* Has any member of your household ever participated in a clinical trial at CERMEL?

0 No 1 Yes 99 NA/DK

O10 *If A6=1 & O9=1* How many members of your households have participated in a clinical trial at CERMEL?

No:___________

Z3D Could we have your phone number?

____

Thank you very much for your time and participation.

Please fill out the following questions without the interviewed person

B1 *If A6=1* Observe the main material of the roof of the dwelling and indicate above:

- 1 Natural roofing
  - 1.1 No roof
  - 1.2 Thatch/Palm leaf
  - 1.3 Sod
- 2 Rudimentary roofing
  - 2.1 Rustic mat
  - 2.2 Palm/bamboo
  - 2.3 Wood planks
  - 2.4 Cardboard
- 3 Finished roofing
  - 3.1 Metal
  - 3.2 Wood
  - 3.3 Calamine/Cement fiber
  - 3.4 Ceramic tiles
  - 3.5 Roofing shingles
- 4 Other: _________________________________
