# Supplementary material for: The economic burden of loiasis: A comprehensive cost-of-illness analysis of regionally representative, individual-level data from rural Gabon
Source: PLoS One. 2026 Feb 23;21(2):e0340689. doi: 10.1371/journal.pone.0340689 (PMC12928485; doi:10.1371/journal.pone.0340689)
Supplement: Attachment — Submitted filename: Response_to_Reviewers.pdf [file pone.0340689.s020.pdf]

Re-submission of:

The economic burden of loiasis: a comprehensive cost-of-illness analysis of regionally representative, individual-level data from rural Gabon

Responses to the academic editor and reviewers

December 06, 2025

Dear academic editor Mr. David T. Zhu,

We thank you for the opportunity to revise and resubmit our manuscript to PLOS ONE. Moreover, we would like to express our gratitude to both reviewers and you for the time taken to review our manuscript and for the constructive comments everyone offered.

In our revised re-submission, we have sought to address each reviewer's insightful comments. Ultimately, we believe that the revised version of the manuscript constitutes a stronger contribution to the field.

In what follows, we detail two main changes we implemented on the manuscript before providing a line-by-line response to each of the reviewers and you. Please note that we refer to reviewers in terms of the journal's numbering: Reviewer #1 and Reviewer #2.

1. Additional analyses: both reviewers provided valuable input on how to further explore and underscore the robustness of our results. In response to these comments, we expanded our robustness checks in multiple ways. We briefly summarize these as follows:

    a. Measurement of loiasis (I): RAPLOA positive vs. RAPLOA negative

    b. Measurement of loiasis (II) Migratory vs. non-migratory loiasis

    c. Alternative model specification: Controlling for respondents' chronic diseases

    d. Heterogeneous effects (I): Impact of loiasis on different socio-economic groups

    e. Heterogenous effects (II) Impact of loiasis by work status

    f. Sensitivity analysis around the uncertainty of parameter estimates used in the extrapolation of Gabon-wide costs

2. Clarifications and precision in the write-up: both reviewers pointed out a number of imprecisions and lacking explanations in our manuscript. We took all of these comments very seriously and aimed to address them. We believe that the revised manuscript is now more concise.

Responses to the academic editor

1. Thank you for submitting your manuscript to PLOS ONE. After careful consideration, we feel that it has merit but does not fully meet PLOS ONE's publication criteria as it currently stands. Therefore, we invite you to submit a revised version of the manuscript that addresses the points raised during the review process. When you resubmit your manuscript, please include line numbering and a point-by-point response to the reviewers indicating which changes you made and on which lines.

   We would like to thank the academic editor for giving us this opportunity. As requested, we have modified the manuscript by including line numbering. In addition, we have prepared a point-by-point response letter to the academic editor and reviewers.

2. If you would like to make changes to your financial disclosure, please include your updated statement in your cover letter.

   We would like to keep the financial disclosure made available in the first version of the submitted manuscript, which states the following (lines 50 – 52):

   "Funding to support this research was provided by the Bernhard Nocht Institute for Tropical Medicine (BNITM) and the German Center for Infection Research (FKZ: 8008803909)."

3. Guidelines for resubmitting your figure files are available below the reviewer comments at the end of this letter.

   We thank you for sharing this information.

4. If applicable, we recommend that you deposit your laboratory protocols in protocols.io to enhance the reproducibility of your results. Protocols.io assigns your protocol its own identifier (DOI) so that it can be cited independently in the future. For instructions see: https://journals.plos.org/plosone/s/submission-guidelines#loc-laboratory-protocols. Additionally, PLOS ONE offers an option for publishing peer-reviewed Lab Protocol articles, which describe protocols hosted on protocols.io. Read more information on sharing protocols at https://plos.org/protocols?utm_medium=editorial-email&utm_source=authorletters&utm_campaign=protocols.

   We will upload the laboratory protocol in due time. Please be assured that it is available.

5. Please ensure that your manuscript meets PLOS ONE's style requirements, including those for file naming. The PLOS ONE style templates can be found at https://journals.plos.org/plosone/s/file?id=wjVg/PLOSOne_formatting_sample_main_body.pdf and https://journals.plos.org/plosone/s/file?id=ba62/PLOSOne_formatting_sample_title_authors_affiliations.pdf

We thank you for sharing this useful information. The manuscript has been adapted accordingly and hopefully now meets all PLOS ONE's style requirements.

6. Please include a complete copy of PLOS' questionnaire on inclusivity in global research in your revised manuscript. Our policy for research in this area aims to improve transparency in the reporting of research performed outside of researchers' own country or community. The policy applies to researchers who have travelled to a different country to conduct research, research with Indigenous populations or their lands, and research on cultural artefacts. The questionnaire can also be requested at the journal's discretion for any other submissions, even if these conditions are not met. Please find more information on the policy and a link to download a blank copy of the questionnaire here: https://journals.plos.org/plosone/s/best-practices-in-research-reporting. Please upload a completed version of your questionnaire as Supporting Information when you resubmit your manuscript.

   We thank you for this suggestion. The questionnaire has been filled in and will be submitted along with the other documents.

7. We note that the grant information you provided in the 'Funding Information' and 'Financial Disclosure' sections do not match. When you resubmit, please ensure that you provide the correct grant numbers for the awards you received for your study in the 'Funding Information' section.

   We would like to thank you and apologize for this inconsistency. We have adjusted the 'Funding Information' and 'Financial Disclosure' as necessary.

8. Thank you for stating the following financial disclosure: "The author(s) received no specific funding for this work."
   At this time, please address the following queries:
   a) Please clarify the sources of funding (financial or material support) for your study. List the grants or organizations that supported your study, including funding received from your institution.
   b) State what role the funders took in the study. If the funders had no role in your study, please state: "The funders had no role in study design, data collection and analysis, decision to publish, or preparation of the manuscript."
   c) If any authors received a salary from any of your funders, please state which authors and which funders.
   d) If you did not receive any funding for this study, please state: "The authors received no specific funding for this work."

   Please include your amended statements within your cover letter; we will change the online submission form on your behalf.

   We would like to thank you for this advice. We included the requested information and clarification in the updated cover letter. Moreover, we provide this information here now as well:
   Please find below a response to each of the listed points:
   a) Funding to support this research was provided by the Bernhard Nocht Institute for Tropical Medicine (BNITM) and the German Center for Infection Research (FKZ: 8008803909).

b) *The funders had no role in study design, data collection and analysis, decision to publish, or preparation of the manuscript.*
c) *The following authors received a salary from BNITM: Cédric Isaac Mbavu, Kerstin Perlik, Rella Zoleko-Manego, Michael Ramharter and Jan Priebe.*
d) *Not applicable.*

9. When completing the data availability statement of the submission form, you indicated that you will make your data available on acceptance. We strongly recommend all authors decide on a data sharing plan before acceptance, as the process can be lengthy and hold up publication timelines. Please note that, though access restrictions are acceptable now, your entire data will need to be made freely accessible if your manuscript is accepted for publication. This policy applies to all data except where public deposition would breach compliance with the protocol approved by your research ethics board. If you are unable to adhere to our open data policy, please kindly revise your statement to explain your reasoning and we will seek the editor's input on an exemption. Please be assured that, once you have provided your new statement, the assessment of your exemption will not hold up the peer review process.

   Yes, we confirm that we will make our data available upon acceptance of the manuscript. Furthermore, we are familiar with posting such data and replication files on public repositories. Lastly, we already have finalized our data sharing plan but would be open to adjust the plan in case of special requirements by the journal.

10. Please include your full ethics statement in the 'Methods' section of your manuscript file. In your statement, please include the full name of the IRB or ethics committee who approved or waived your study, as well as whether or not you obtained informed written or verbal consent. If consent was waived for your study, please include this information in your statement as well.

   Thank you for this advice. We acknowledge that the previous ethics statement was already present in the 'Methods' section but was not yet complete. In response, we added the additional content in the respective section paragraph.

11. We note that Figure 1 in your submission contain map/satellite images which may be copyrighted. All PLOS content is published under the Creative Commons Attribution License (CC BY 4.0), which means that the manuscript, images, and Supporting Information files will be freely available online, and any third party is permitted to access, download, copy, distribute, and use these materials in any way, even commercially, with proper attribution. For these reasons, we cannot publish previously copyrighted maps or satellite images created using proprietary data, such as Google software (Google Maps, Street View, and Earth). For more information, see our copyright guidelines: http://journals.plos.org/plosone/s/licenses-and-copyright.

   We require you to either (1) present written permission from the copyright holder to publish these figures specifically under the CC BY 4.0 license, or (2) remove the figures from your submission:

   a. You may seek permission from the original copyright holder of Figure 1 to publish the content specifically under the CC BY 4.0 license.

We recommend that you contact the original copyright holder with the Content Permission Form (http://journals.plos.org/plosone/s/file?id=7c09/content-permission-form.pdf) and the following text:

“I request permission for the open-access journal PLOS ONE to publish XXX under the Creative Commons Attribution License (CCAL) CC BY 4.0 (http://creativecommons.org/licenses/by/4.0/). Please be aware that this license allows unrestricted use and distribution, even commercially, by third parties. Please reply and provide explicit written permission to publish XXX under a CC BY license and complete the attached form.”

Please upload the completed Content Permission Form or other proof of granted permissions as an "Other" file with your submission.

In the figure caption of the copyrighted figure, please include the following text: “Reprinted from [ref] under a CC BY license, with permission from [name of publisher], original copyright [original copyright year].”

b. If you are unable to obtain permission from the original copyright holder to publish these figures under the CC BY 4.0 license or if the copyright holder’s requirements are incompatible with the CC BY 4.0 license, please either i) remove the figure or ii) supply a replacement figure that complies with the CC BY 4.0 license. Please check copyright information on all replacement figures and update the figure caption with source information. If applicable, please specify in the figure caption text when a figure is similar but not identical to the original image and is therefore for illustrative purposes only.

The following resources for replacing copyrighted map figures may be helpful:

USGS National Map Viewer (public domain): http://viewer.nationalmap.gov/viewer/

The Gateway to Astronaut Photography of Earth (public domain): http://eol.jsc.nasa.gov/sseop/clickmap/

Maps at the CIA (public domain): https://www.cia.gov/library/publications/the-world-factbook/index.html and https://www.cia.gov/library/publications/cia-maps-publications/index.html

NASA Earth Observatory (public domain): http://earthobservatory.nasa.gov/

Landsat: http://landsat.visibleearth.nasa.gov/

USGS EROS (Earth Resources Observatory and Science (EROS) Center) (public domain): http://eros.usgs.gov/#

Natural Earth (public domain): http://www.naturalearthdata.com/

We thank you for these comments and suggestions. Figure 1 (Panel A) plots the location of study villages on an administrative map of Gabon provided by GADM. GADM allows the use of

their data to create maps and figures for publishing academic research. We did not use any licensed satellite images. Therefore, this content can be published under the Creative Commons Attribution License (CC BY 4.0).

12. Please include captions for your Supporting Information files at the end of your manuscript, and update any in-text citations to match accordingly. Please see our Supporting Information guidelines for more information: http://journals.plos.org/plosone/s/supporting-information.

    We thank you for this comment. We included the captions at the end of the manuscript for the supporting information and updated the text accordingly.

13. If the reviewer comments include a recommendation to cite specific previously published works, please review and evaluate these publications to determine whether they are relevant and should be cited. There is no requirement to cite these works unless the editor has indicated otherwise.

    None of the reviewers recommended to cite specific previously published works.

14. Please review your reference list to ensure that it is complete and correct. If you have cited papers that have been retracted, please include the rationale for doing so in the manuscript text, or remove these references and replace them with relevant current references. Any changes to the reference list should be mentioned in the rebuttal letter that accompanies your revised manuscript. If you need to cite a retracted article, indicate the article's retracted status in the References list and also include a citation and full reference for the retraction notice.

    We thank you for this valuable contribution. We reviewed the references list and ensured that it is complete and correct. We made one formal change to the sources by adding the names "Alleyne GA, Cohen D" in the publication on "Health, economic growth, and poverty reduction" by the WHO (line 704).

    Two references have been deleted:

- *The former reference [21]: WHO. ESPEN 2019 Annual Report. World Health Organization, 2019.*
- *The former reference [33]: Group WB. World Development Indicators 2023 [cited 2025 05 March]. Available from: https://databank.worldbank.org/source/world-development-indicators.*

Two other references have been added:

- *Reference [12]: Ramharter M, Schlabe S, Hubner MP, Michelitsch P, Kurth F, Belard S, et al. Diagnosis, management and prevention of loiasis: guideline of the German Society for Tropical Medicine, Travel Medicine, and Global Health (DTG). Infection. 2025;53(3):851-72. Epub 20250521. doi: 10.1007/s15010-024-02443-2. PubMed PMID: 40397272; PubMed Central PMCID: PMCPMC12137371.*
- *Reference [20]: Kelly-Hope LA, Hemingway J, Taylor MJ, Molyneux DH. Increasing evidence of low lymphatic filariasis prevalence in high risk Loa loa areas in Central and West Africa: a*

literature review. Parasit Vectors. 2018;11(1):349. Epub 20180615. doi: 10.1186/s13071-018-2900-y. PubMed PMID: 29907117; PubMed Central PMCID: PMCPMC6004093.

Lastly, we did not cite papers that have been retracted.

Responses to Reviewer #1

1. The article by Mbavu et al. reports a study on the economic impact of loiasis in Gabon. This is likely the first study addressing this question, and although the approach is somewhat partial, it merits publication.

   We very much appreciate Reviewer #1's assessment.

2. There are no line numbers, which makes referencing specific comments difficult.

   We apologize for this negligence. For this revision we now have adjusted the manuscript and added line numbers. We hope that this addresses Reviewer #1's concerns.

3. Some references are unclear or not entirely appropriate—for example, references 12–15 for Gabon (Takougang and Pinder seem not well suited). This occurs in several parts of the manuscript. Similarly, the introduction lacks precision: it is unclear which references support the statement about USD 58. Shorter, clearer sentences and precise citations would help readers unfamiliar with loiasis to follow the logic and evidence.

   Thank you for your comment. Moreover, we agree with your assessment. Consequently, we carefully reviewed all references to ensure that each is correctly placed within the manuscript, and we also revised several sentences to improve readability. We hope that the revised manuscript addresses Reviewer #1's concerns adequately.

4. The manuscript also lacks the ethical approval number and any administrative authorization.

   We admit that we were unaware that this detailed information was required in the main manuscript text. We thank Reviewer #1 for pointing this out to us. We now have included the approval number of the responsible ethics committee in the revised version of the manuscript. The study was approved by the institutional Scientific Review Committee and authorized by the Board of Directors of CERMEL.

5. Regarding sampling, beyond the total target of 1,100 individuals, it is not clear if there was a minimum number of participants per village or a minimum proportion required.

   Thank you very much for this comment. We did not have any minimum number of participants per village. We aimed to reach every adult (aged between 18 and 65 years old) in each village. In response to your comment, we included a sentence in the manuscript to clarify this (line 149). We hope that this addresses Reviewer #1's concern.

6. Concerning loiasis status, some results suggest different roles for microfilariae versus adult worms. Perhaps it would have been useful to include a sensitivity analysis using the same methodology applied to only mf+ vs mf- and RAPLOA+/mf- vs RAPLOA-/mf-. Adding this or discussing it in the discussion could provide additional insight.

   Thank you very much for this excellent comment.

   Following your suggestion, we have performed further analyses using alternative measures of loiasis status to account for the possibly different effects of microfilariae and adult worms. First, we compared RAPLOA positive to RAPLOA negative individuals (regardless of their microfilaremia status) (S7 Table). Second, in line with the most nascent research on loiasis pathophysiology, we compared migratory to non-migratory loiasis participants (S8 Table). This classification is described in detail by Ramharter et al. (2025) and refers to patients that exhibit symptoms such as eye worm migration and Calabar swelling, regardless of their microfilaremia status (migratory loiasis patients), as opposed to individuals who are microfilaremia positive, but without any signs of adult worm migration (non-migratory loiasis individuals). This classification is considered particularly relevant by clinicians.

   The additional sensitivity analyses reveal that the cost of the disease is mainly driven by RAPLOA positive individuals, with a significant difference in both analyses for the indirect costs. This new piece of information is added in the manuscript at lines 289 to 293.

7. From a methodological perspective, I am not an expert in health economics, but several points are worth noting. The study uses a solid approach with entropy balancing and generalized linear models (GLM), which is generally accepted in cost-of-illness studies. The distinction between direct medical, direct non-medical, and indirect costs is clear. Including both formal and informal healthcare expenditures, such as traditional healers, is particularly valuable given the local context. Using national health insurance data (CNAMGS) to validate self-reported expenditures and converting to USD adds transparency. However, there are some limitations. The cross-sectional design only captures short-term costs and cannot assess causality. Chronic or secondary complications, such as renal problems, neurological effects, or other organ dysfunctions, are not included, which may underestimate the total societal burden. It would be helpful for the authors to clarify whether the goal was to measure short-term individual costs or broader societal costs, and especially to discuss how long-term complications might change the estimates.

   We would like to thank Reviewer #1 for this comment. In the revised manuscript, we clarify and argue that our cost estimates relate to a societal perspective (not only individual costs). We refrain from referring to the estimates as short or long-term estimates since the years since infection cannot be traced in our context. Furthermore, as part of the discussion section we flag the limitation that our estimates are (i) not causal and (ii) do only control for a limited set of co-morbidities. As discussed below under point 10, we believe that our estimates are not driven by comorbidities. We hope that these adjustments address Reviewer #1's concerns in full.

8. The indirect cost estimates rely on self-reported data on days missed or reduced productivity, which may not be precise. It is also unclear how the authors assigned a monetary value to the work of participants without a fixed salary, such as subsistence farmers.

   This is a point well-taken. First, we acknowledge the caveat related to self-reported data and discuss this as a limitation of our study at lines 478 – 489.

   Second, with respect to the monetary value of absenteeism and presenteeism (for the entire sample, without distinction of work status), we estimate an average daily wage using the following strategy: during the interviews, we collected data on the monetary value of agricultural production and any sort of in-kind income (e.g., selling woods or herbs from the forest or own consumption of harvested agricultural products). In addition, we asked each household to report their financial income and any financial assistance (e.g., from family members) they received during the month prior to the interview. We then divided this total income (for each household) by the number of individuals who can participate in income generating activities in that household, i.e., individuals older than 10 years of age. We calculated the average of this parameter over the entire sample. This average parameter for all households was then divided by 24 (excluding the weekends), in order to obtain the daily wage. Finally, this daily wage was used to estimate the value of missed working days and reduced productivity. We included this description in S15 Text.

9. Additionally, it would help to clarify whether these costs are calculated from the perspective of the individual, the household, or society as a whole.

   This is an important point since it has strong implications for the interpretation of our results. We would like to apologize that the previous version of the manuscript did not state this clearly enough. We now highlight more explicitly that we adopted a societal perspective. This is now referenced from line 81 to line 88 in the manuscript.

   Though you have not explicitly asked for this, we briefly explain the rationale behind this approach. Following Jo's (2014) classification, we gathered comprehensive data on direct medical costs, direct non-medical costs, and indirect costs. This approach allowed us to capture a wide range of expenses that extend beyond resources used solely by the healthcare system, government, or third-party payers. By doing so, we obtained a holistic view of the burden of loiasis, including productivity losses. Considering the high prevalence of loiasis in the region—nearly half of the population is affected—, a cost-of-illness analysis from a societal perspective was deemed the most appropriate method.

10. Another concern is comorbidities. While malaria is included as a covariate, other common conditions like Mansonella perstans or chronic diseases may also affect both infection and healthcare costs. Clarifying whether these were considered would strengthen the analysis.

    We thank Reviewer #1 for this comment and suggestion.

    Indeed, we cannot fully rule out that (i) individuals might have an additional particular co-infection (or alternative underlying illness) and that (ii) such a co-infection might influence our estimates. In our view, this is a frequent constraint of these type of studies in LMICs, as very

often one cannot collect biomarkers on all possible illnesses. We acknowledge this limitation explicitly towards the end of the manuscript (line 489 to line 504).

However, we are able to control for self-reported illnesses in our analysis. Albeit self-reported data can be unreliable, we at least can partially mitigate concerns that our estimates are driven by co-morbidities. More specifically, we now added an additional table (S9 Table) in which we control for self-reported underlying chronic illnesses. We found that our main results seem not to be affected by the inclusion of these variables. Although we could not control explicitly for Mansonella perstans (as we did not test all the participants for it), we believe that our results are not much affected by co-morbidities.

11. Differences in cost distribution by gender or wealth could also be explored.

    Thank you very much for your suggestion. We have included your proposed additional analyses. With respect to the dimension of gender (see Table 3, lines 333-338 in the manuscript), we find that costs related to loiasis are higher for women compared to men.

    For the heterogeneity by wealth, we use a PCA-derived wealth index based on respondents' answers to questions about their household characteristics (such as access to electricity, a radio, a television, a decoder, a mobile phone and a certain type of roof) and generated two groups: poorer and richer individuals divided by the median of the wealth of the household. This analysis reveals that while poorer individuals incur more direct medical costs, i.e. 43.74 US Dollar per year (significant at 1 percent level), wealthier individuals bear higher non-medical and indirect costs (26.34 USD and 82.76 USD, respectively; p-value < 0.001 for both categories) (S11 Table & lines 338 – 341 in the manuscript). We hope that these additional analyses address Reviewer #1's comments.

12. The authors tested the robustness of their GLM results by comparing different distributions (gamma vs. negative binomial) and alternative covariate specifications. This is reassuring, although it would be useful to briefly comment on whether these alternatives produced notably different results or just minor changes.

    Thank you very much for your comment. Overall, the changes made to the model specification do not produce notable differences. We included this information at lines 287-289.

13. Village fixed effects are included, but potential spatial correlation between villages is not discussed.

    Thank you very much for the comment. We include village fixed effects in our estimation to control for a broad set of local characteristics that could bias our result. The identification thus comes from comparing persons within a village. Given that the selected villages are geographically all quite close, including correction of spatial standard error clusters or other tools are not appropriate from our point of view. Likewise, the overall number of villages is still rather small in the study region so that some common statistical adjustment procedures (e.g., Conley-type standard error adjustments) would suffer from efficiency concerns.

To assess whether there are any spatial clusters in our main variables, we instead show the geographical distribution of those variables in S10 Figure. The figure shows on the left the average share of loiasis positive individuals per village and on the right the average health costs per village. Looking at both maps, we cannot find any spatial clusters within the narrowly selected study area. Based on this visual inspection, we conclude that it is rather unlikely the spatial correlations in the incidence of loiasis that affect our main estimates.

14. How hospital and outpatient costs were constructed at the unit level could also be clarified: were expenditures broken down by consultations, diagnostics, medications, etc.?

    Thank you very much for your comment. Following your advice, we have included further information on the construction of inpatient and outpatient costs in the manuscript - see line 182 to line 191. Specifically, we collected detailed information on how much participants spent for each of the following categories at their last stay (in inpatient care) and/or last visit (outpatient care): consultation, medication, diagnostic tests, medical devices, documentation, accommodation, informal payments, transportation, as well as their waiting time and the time spent by caregivers helping during the sickness.

    We also asked about the number of times they had been in inpatient and/or outpatient care over the previous 3 months. Based on this information, we extrapolated the cost to one quarter by multiplying the cost of one stay (or visit) by the number of stays (or visits) in this 3-month period. Finally, the total yearly costs were found by multiplying the quarterly costs by 4.

15. Overall, the methodology is strong but could benefit from a clearer explanation of perspective, more details on productivity loss measurement, and discussion of cross-sectional limitations and excluded chronic complications.

    Thank you very much for your comment. Following your advice, we have included more details with respect to the perspective and construction of measures, as well as the limitations of our study. We will explain briefly the changes made.

    With respect to the perspective, our cost-of-illness study emphasizes a societal perspective (see point 9 above).

    *With respect to the measurement of productivity losses, we consider two main aspects: absenteeism and presenteeism. Absenteeism was measured by asking the respondents the number of days they were not able to work because of health problems. Our estimation of presenteeism relies on a short form of the Health and Labor Questionnaire (Van Roijen et al., 1996). Using the Osterhaus's method, we asked two questions to the respondents: 1) The number of days they work although bothered by health problems; 2) An evaluation of their performance at work from 0 (much worse than usual) to 10 (similar performance as usual). The productivity loss was determined by multiplying the number of days worked less efficiently by one minus the efficiency for these days. For instance, a hypothetical individual reported that he/she was bothered by health problems for two days. He/she declared a performance of five. The productivity loss will be: 2 * (1-0.5) = 1 workday. This lost workday will then be multiplied by the daily income to generate a monetary value of productivity loss for presenteeism. We included this information in S14 Text.*

With respect to chronic diseases, in our main analysis, we have not excluded participants that report to have chronic diseases. Instead, we conducted further sensitivity analysis by adding 'chronic diseases' as a covariate in our model. During the interviews, we asked participants if they had any chronic diseases. Only 179 participants (15 percent) in our sample declared having at least one. Adding the presence of 'chronic diseases' (a binary variable) as a covariate did not substantially change the pattern and magnitude of costs. The results of this analysis are displayed in S9 Table.

Lastly, we acknowledge and discuss the limitations of cross-sectional study designs. Indeed, due to limited available resources, we did not follow our participants over time. This limitation is extensively discussed in the manuscript from line 478 to 489.

16. Results and discussion are generally clear. The presentation of both aggregate and stratified analyses is helpful. However, the interpretation of USD 40 annual additional cost per person could be clarified: does it include only short-term costs, or could longer-term complications change the estimate?

    The estimate includes both short-term and long-term costs since the onset of loiasis could not be determined based on the questionnaire screening tool (RAPLOA) and microfilaremia diagnostic. We, therefore, abstain from interpreting the results as referring to short or long-term effects.

    However, we now added in the discussion section a paragraph that outlines how we think about its interpretation with respect to longer-term complications (see line 452 to 459).

17. The higher costs observed among women and younger adults are interesting, but alternative explanations such as labor patterns or care-seeking behavior could also be discussed.

    We would like to thank Reviewer #1 for this valuable comment. We conducted more analysis to compare the impact of loiasis on different groups of respondents. Considering their working status, we split our sample into (i) individuals that work and (ii) those without any income activity. Working individuals include employees with a formal employment status, but also informal workers and self-employed (e.g. in agriculture or fishery). We find an increased marginal cost of loiasis on indirect costs, particularly for individuals who are not working (53.21 US Dollar per year, p-value < 0.05). On the other hand, direct non-medical costs are higher for workers, but to a lesser extent (8.97 US Dollar per year, p-value < 0.01). These results highlight the negative effect of the disease on labor market participation (S12 Table).

18. The discussion on including loiasis in insurance packages is relevant. Extrapolation to national costs is reasonable but should account for uncertainty; sensitivity ranges would help.

    This is a very valid point. We updated the respective paragraph to more explicitly describe for which parameters we conduct sensitivity checks to account for the uncertainty of parameter estimates. More specifically, we address uncertainty with respect to three main parameters:

the prevalence rate of loiasis in both rural and urban areas, and the rural vs. urban price ratio. The presented ranges for the main cost estimate are derived from bootstrapping procedures using uniform densities for the underlying plausibility ranges.

19. Limitations are well acknowledged, but endogeneity between infection status and health costs could be discussed further.

    In our study design and analysis, we made use of two approaches to reduce any bias and generate reliable estimates of health-related costs. First, by conducting a large-scale survey in the region of interest and randomly selecting all available respondents, we reduced as much as possible the selection bias. Second, given that our data come from an observational study, we implemented a matching reweighing technique to produce two comparable groups. Based on observable characteristics such as age, gender, wealth status, a proxy for health status (malaria infection), we were able to balance the loiasis negative group and make it similar to the loiasis positive group, with one single difference related to their loiasis infection status. We believe that we contained the risk of endogeneity by doing so. Nevertheless, we cannot completely rule out the issue of omitted variable bias, even though we controlled for a large set of variables that have an impact on our outcomes of interest.

20. In summary, this study provides valuable empirical evidence on the economic burden of loiasis using appropriate methods for a cost-of-illness study and deserves to be published. Its main limitations are the short-term focus, reliance on self-reported data, and exclusion of severe complications. Including discussion or estimates of these additional costs, and clarifying perspective and measurement details, would strengthen interpretation and policy relevance.

    We would like to thank Reviewer #1 for the positive evaluation of our manuscript. In the previous comments, we have addressed specifically the requested issues on time horizon, self-reported data, and exclusion of severe complications. We hope that our responses and changes made are satisfactory for the reviewer.

Responses to Reviewer #2

1. The manuscript is on an important topic for the disease of concern. In general, the manuscript is well written and easy to follow. However, I do have some minor comments:

   We would like to thank Reviewer #2 for this assessment.

2. The funding statement has to be consistent throughout the manuscript: Sometimes no funding, and sometimes there is funding. Please proceed to update the manuscript throughout.

   We apologize for this inconsistency. The manuscript has been revised and contains clear information about the funding. We believe the revised manuscript addresses all these concerns.

3. In the introduction section, page #2, the following statement (According to the authors' preferred specifications, loiasis resulted in treatment costs of 58 United States Dollar (USD) per person per year) needs a reference:

   We apologize for this omission. We have included the requested reference, namely a publication by Veletzky et al. (2024).

4. In the core manuscript, the ethical approval number is missing, while this is mentioned in the submission system.

   We apologize for this mistake. We have added the ethical number in the core manuscript, as recommended (see line 133).

5. Figure 1, Panel A The map can be included in the Country Gabon map, including the names of localities, if possible.

   We appreciate this suggestion made by Reviewer #2. To ease the localization of the villages in the country, we produced a new, larger map that displays the entire country (i.e., Gabon). We hope that this will meet the expectations of Reviewer #2.

6. To better weigh the burden of the additional cost of Loaisis on health expenses, it will be interesting for the author to report on the average annual income of the population of interest, including any in-kind and cash income, if available.

   We thank Reviewer #2 for this comment. In the manuscript, at line 249, the monthly per capita income is reported (which is 68.73 US Dollar). This amount was derived from the cash and in-kind income reported by the respondents during the survey.
